# Mobile Colistin-Resistant Genes *mcr-1*, *mcr-2*, and *mcr-3* Identified in Diarrheal Pathogens among Infants, Children, and Adults in Bangladesh: Implications for the Future

**DOI:** 10.3390/antibiotics13060534

**Published:** 2024-06-07

**Authors:** Shafiuzzaman Sarker, Reeashat Muhit Neeloy, Marnusa Binte Habib, Umme Laila Urmi, Mamun Al Asad, Abu Syed Md. Mosaddek, Mohammad Rabiul Karim Khan, Shamsun Nahar, Brian Godman, Salequl Islam

**Affiliations:** 1 Department of Microbiology, Jahangirnagar University, Savar, Dhaka 1342, Bangladesh; shafiuzzamansarker123@gmail.com (S.S.); reeashat.neeloy@gmail.com (R.M.N.); marnusamomo@gmail.com (M.B.H.); m.urmi@unsw.edu.au (U.L.U.); ronniebge22@gmail.com (M.A.A.); nahar@juniv.edu (S.N.); 2 School of Optometry and Vision Science, UNSW Sydney, Sydney, NSW 2052, Australia; 3 Department of Pharmacology, Uttara Adhunik Medical College, Dhaka 1230, Bangladesh; drmosaddek1968@gmail.com; 4 Sheikh Hasina National Institute of Burn and Plastic Surgery (SHNIBP), Dhaka 1000, Bangladesh; rabiulpapon77@yahoo.com; 5 Strathclyde Institute of Pharmacy and Biomedical Sciences, University of Strathclyde, Glasgow G4 0RE, UK; brian.godman@strath.ac.uk; 6 Division of Public Health Pharmacy and Management, School of Pharmacy, Sefako Makgatho Health Sciences University, Pretoria 0204, South Africa

**Keywords:** mobile colistin resistance, *mcr* gene, human-*mcr*, diarrheal infant patients, Bangladesh, MDR, antimicrobial stewardship programs

## Abstract

Colistin is a last-resort antimicrobial for treating multidrug-resistant Gram-negative bacteria. Phenotypic colistin resistance is highly associated with plasmid-mediated mobile colistin resistance (*mcr*) genes. *mcr*-bearing *Enterobacteriaceae* have been detected in many countries, with the emergence of colistin-resistant pathogens a global concern. This study assessed the distribution of *mcr-1*, *mcr-2*, *mcr-3*, *mcr-4*, and *mcr-5* genes with phenotypic colistin resistance in isolates from diarrheal infants and children in Bangladesh. Bacteria were identified using the API-20E biochemical panel and 16s rDNA gene sequencing. Polymerase chain reactions detected *mcr* gene variants in the isolates. Their susceptibilities to colistin were determined by agar dilution and E-test by minimal inhibitory concentration (MIC) measurements. Over 31.6% (71/225) of isolates showed colistin resistance according to agar dilution assessment (MIC > 2 μg/mL). Overall, 15.5% of isolates carried *mcr* genes (7, *mcr-1*; 17, *mcr-2*; 13, and *mcr-3*, with co-occurrence occurring in two isolates). Clinical breakout MIC values (≥4 μg/mL) were associated with 91.3% of *mcr*-positive isolates. The *mcr*-positive pathogens included twenty *Escherichia* spp., five *Shigella flexneri*, five *Citrobacter* spp., two *Klebsiella pneumoniae*, and three *Pseudomonas parafulva*. The *mcr*-genes appeared to be significantly associated with phenotypic colistin resistance phenomena (*p* = 0.000), with 100% colistin-resistant isolates showing MDR phenomena. The age and sex of patients showed no significant association with detected *mcr* variants. Overall, *mcr*-associated colistin-resistant bacteria have emerged in Bangladesh, which warrants further research to determine their spread and instigate activities to reduce resistance.

## 1. Introduction

Emerging antimicrobial resistance (AMR) is a significant public health concern [1]. In 2019, it was estimated there were 4.95 million deaths globally associated with bacterial AMR, including 1.27 million deaths directly attributable to bacterial AMR, with the highest mortality currently seen in South Asian and sub-Saharan African countries [2]. In addition, considerable morbidity and costs are associated with AMR [3,4,5,6], with AMR driven by the overuse and misuse of antibiotics [7,8,9,10]. While AMR is a universal phenomenon, the burden among low- and middle-income countries (LMICs) is appreciably higher due to economic, political, and environmental factors, including poor governance and infrastructures, as well as a limited number of national initiatives [11,12,13,14]. This is now changing with global initiatives, including the Global Action Plan (GAP) by the World Health Organization (WHO) to reduce AMR [1] and others from the World Bank and OECD [6,15]. The GAP has been translated into National Action Plan (NAP), which includes Bangladesh, with LMICs at different stages of their implementation due to resource and other issues [16,17,18,19].

Other important global initiatives include dividing antibiotics into three different categories based on their resistance potential, which includes the ‘Access’, ‘Watch’, and ‘Reserve’ categories [20,21]. Antibiotics in the ‘Reserve’ category, which include fifth-generation cephalosporins, some carbapenems, and linezolid, should only be prescribed in multidrug resistance cases, with the aim of curbing rising AMR rates [20,22,23,24].

Colistin is also classified as a ‘Reserve’ antibiotic following the global increase in the prevalence of carbapenem-resistant *Enterobacteriaceae* [21,25], with concerns with its overuse and resultant resistance development [26,27]. Concerns with its overuse in both animals and humans, with resultant resistance development via zoonotic gene transfers, coupled with its importance in treating resistant Gram-negative infections to reduce morbidity and mortality, have resulted in the WHO and others classifying colistin as an antibiotic of very high importance for use in humans, with its use reserved [20,28,29,30,31,32,33,34]. Alongside this, many countries now ban the use of colistin as a growth promoter in animal feeds and prophylactically to prevent bacterial infections [35,36,37], with such measures shown to be effective in reducing resistant strains [36,37,38]. This is important in Bangladesh, given extensive colistin-resistant *Escherichia coli* in broiler meat and chicken feces [39,40,41] exacerbating resistance among patients to colistin in Bangladesh [42,43,44,45]. Over-the-counter dispensing of antibiotics is also common in Bangladesh and is a concern, especially when this involves ‘Reserve’ antibiotics [46,47,48,49]

The use of colistin as an antibiotic of last resort is greatly threatened by its overuse and the associated rise of plasmid-borne mobile colistin resistance genes [50,51,52], spreading rapidly via horizontal gene transfer [53]. Resistance to colistin is generated by the chromosomally mediated modification of lipopolysaccharide (LPS) [54]. The acquisition of colistin resistance by a novel plasmid-mediated gene, *mcr-1*, was first described in *Enterobacteriales* from both farm-animal products and humans [55]. Earlier studies have shown the genotypic linkage of the mobile colistin resistance gene *mcr-1* to phenotypic colistin resistance [56,57]. Since then, variants of *mcr*-carrying multiple species of *Enterobacteriales* have been detected in many countries from environments, animals, and humans [58,59,60,61]. Subsequently, more variants of the transferable colistin resistance *mcr* gene (*mcr-1* to *mcr-9*) have been described in *Enterobacteriaceae* [62,63].

This is a concern. A recent outbreak involving colistin-resistant pathogens in China resulted in a very high case-fatality rate in humans [64]. Consequently, the identification of the root cause, transmission, and trajectories of colistin-resistant infections is an increasing priority globally. We are aware that *mcr*-gene variants can be detected in the environment, animals, human fecal samples, and food products [65]. However, only a limited number of studies have also shown the dissemination of plasmids carrying these variants in infants with acute diarrhea [66]. This is important in Bangladesh, with diarrhea being a major cause of childhood mortality in the country, combined with the increasing prevalence of resistant genes in children, exacerbated by the overuse of antibiotics [67,68,69].

Consequently, this study was designed to investigate different variants of the *mcr* gene and their association with colistin resistance among diarrheal pathogens in infants, children, and adults in Bangladesh, as well as the demographic factors associated with identified *mcr* variants. The findings can be used to suggest future policies and initiatives in Bangladesh and beyond where there are concerns.

## 2. Results

### 2.1. Study Patients

**Figure 1:**
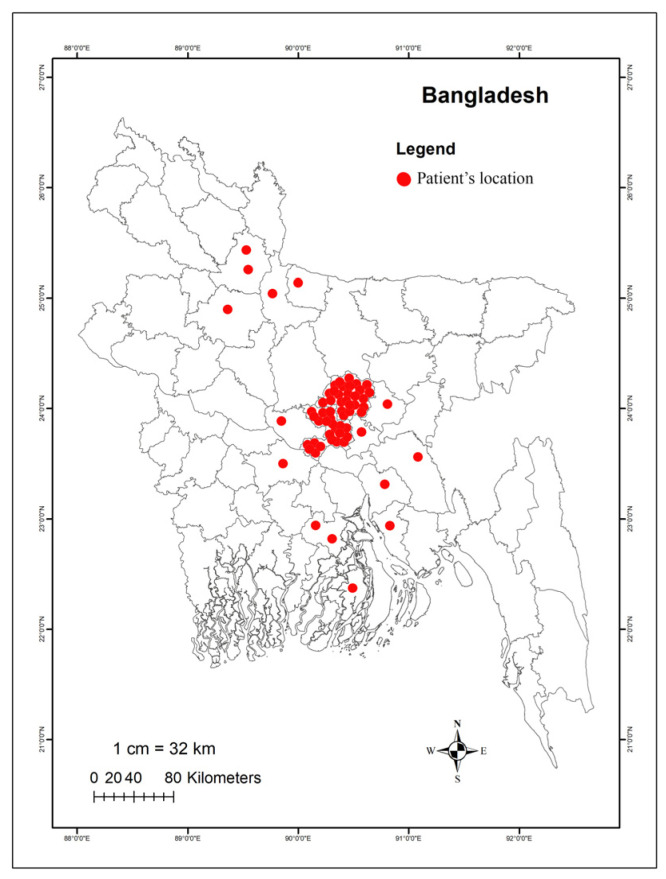
Sampling Location. Different sites are marked, indicating the spatial distribution of diarrheal patients from whom samples were collected. The map displays sampling sites across various cities in Bangladesh.

We collected a total of 179 diarrheal stool samples from infants, children, and adults in different locations in Bangladesh (Figure 1) throughout our study. We subsequently isolated 228 distinct bacteria from 168 culture-positive diarrheal patients. The study patients comprised 102 (57%) males and 77 (43%) females.

**Figure 2:**
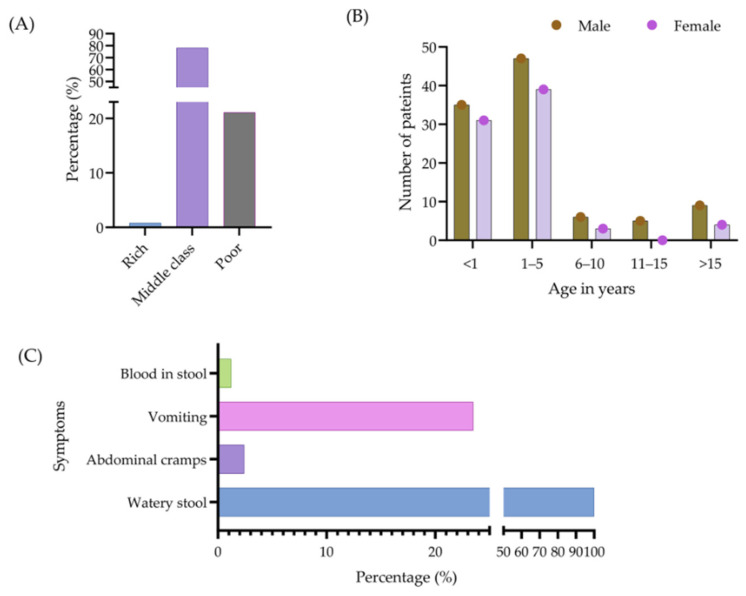
Socioeconomic status of patients (**A**), as well as their age, sex (**B**), and clinical symptoms associated with diarrhea (**C**).

In 11 stool samples, no bacterial growth appeared. The majority of the study patients were infants and children who needed hospitalization (admitted to Uttara Medical College, Dhaka), and the median and interquartile range (IQR) age was 1.17 (0.75–2.5) years. Additionally, 78.1% of patients were middle class, 21.1% were poor, and 0.8% were rich (Figure 2A). The duration of diarrhea among all patients ranged from 3 to 40 days, and the mean duration (standard deviation) was 7.2 ± 4.78 days.

The ages of the patients were categorized into five groups, namely <1 year, 1–5 years, 6–10 years, 11–15 years, and >15 years, with disease occurrence highest in the 1–5 years age group (48%). Figure 2B provides further information regarding the incidence of diarrhea associated with each age group. Among all the patients, 179 (100%) appeared with watery stools, 4 (2.2%) presented with abdominal cramps, 39 (21.8%) with vomiting, and 2 (1.10%) patients with blood in their stools (Figure 2C).

### 2.2. Identification of Diarrheal Pathogens and Their Phenotypic Colistin Susceptibility

**Figure 3:**
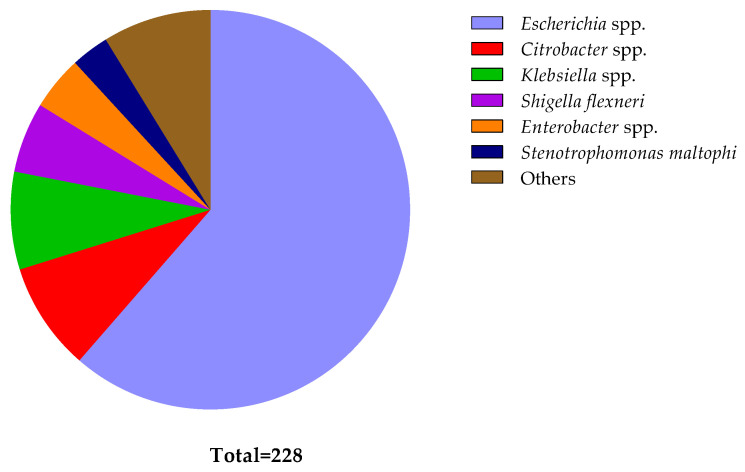
Distribution of different bacteria identified from diarrheal patients. The pie chart illustrates the relative numbers of various bacteria identified among diarrheal patients. Each segment represents a specific bacterial species, with the size of each segment corresponding to the frequency of its occurrence in the sampled population. The ‘Other’ segment comprises bacteria found in singular instances (*Bacillus cereus*, *Bacterium endosymbiont*, *Cronobacter sakazakii*, *Enterococcus faecium*, *Proteus mirabilis*, *Serratia marcescens*, and *Vibrio neocaledonicus*), bacteria found in two (*Acinetobacter* spp. and *Staphylococcus* spp.), and bacteria found in three (*Morganella morganii*, *Aeromonas caviae*, and *Pseudomonas parafulva*).

Of the 228 isolates, 140 were categorized as *Escherichia* spp. (61.40%), 20 as *Citrobacter* spp. (8.77%), 18 as *Klebsiella* spp. (7.89%), 13 as *Shigella flexneri* (5.70%), 10 as *Enterobacter* spp. (4.39%), and 7 as *Stenotrophomonas maltophi* (3.07%). Figure 3 contains details of all the pathogens identified. One *Proteus* sp. and two staphylococci were excluded from further study since colistin resistance is natural in *Proteus* and *Staphylococcus* species [70,71].

The agar dilution method determined the test bacteria as susceptible (S) when there was no growth at ≤2 μg/mL colistin sulfate concentrations and resistant (R) when growth appeared at >2 μg/mL. In addition, the disk diffusion method was used to evaluate the antibacterial potency of colistin sulfate (25 µg) in vitro for the 225 isolates, and bacteria were considered colistin-resistant (R) if ≤10 mm diameter zone of inhibition was recorded (Materials and Methods Section (Section 4)). Among the 225 isolates, the agar dilution test revealed that 69 (30.7%) isolates were resistant to colistin sulfate. However, the colistin disk diffusion method showed that 180 (80.7%) isolates were resistant. Where possible, the results were validated by a minimum inhibitory concentration (MIC) assessment using the gold standard broth microdilution method. Overall, there was over 90% agreement in the results obtained between the agar dilution test and broth microdilution. In view of this, we considered the agar dilution test results as validated and subsequently disregarded the colistin disk diffusion results for subsequent analyses, similar to other authors [72].

### 2.3. Prevalence of mcr Genes in Diarrheal Isolates

All 225 isolates were subjected to polymerase chain reaction (PCR) to find *mcr-1* to *mcr-5* genes. Three types of *mcr* genes (*mcr-1*, *mcr-2*, and *mcr-3*) were detected in 35 isolates. These included 20 for *Escherichia* spp., five for *Shigella flexneri*, five for *Citrobacter* spp., two for *Klebsiella pneumoniae* and *Enterobacter hormaechei*, and one for *Pseudomonas parafulva*. Bacteria identified from 10 other genera did not appear with *mcr* genes (Table 1).

Of the 35 *mcr* positive isolates, co-occurrence was identified in 2 isolates, one containing *mcr-1* and *mcr-2* and the other containing *mcr-2*, and *mcr-3*. The harborages of *mcr-1*, *mcr-2*, and *mcr-3* were identified as 3.1% (7 isolates), 7.6% (17 isolates), and 5.8% (13 isolates), respectively. Combined, the presence of *mcr* variants was 15.56% (35/225).

### 2.4. Phenotypic–Genotypic Association

Of the 35 *mcr*-positive isolates, the agar dilution test identified 32 (91.4%) resistant isolates and 3 (8.6%) sensitive isolates, which revealed a very high statistical significance of *mcr* variant gene associations (*p* = 0.000). Further separate analyses showed very significantly high statistical associations of *mcr-1*, *mcr-2*, and *mcr-3* with phenotypic colistin resistance (*p* = 0.000 for all three gene variants) (Table 2). All of the test isolates grew well on the control plate without colistin sulfate. As a susceptible control, the *Escherichia coli* ATCC 25922 strain with an MIC of 2 µg/mL was employed.

**Figure 4:**
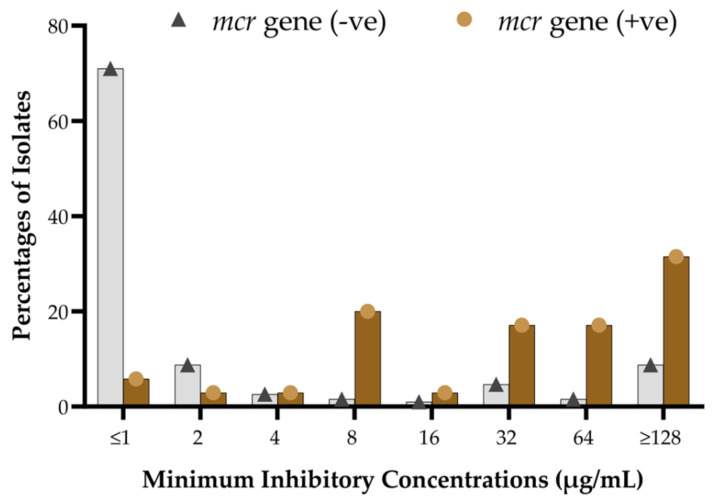
Distribution of MIC levels of colistin sulfate among *mcr*-positive and *mcr*-negative isolates.

The MIC was determined by *E*-test and agar dilution test separately, using a range of ≤0.5 µg/mL to >256 µg/mL (Materials and Methods Section (Section 4)). The median and IQR MIC for *mcr*-positive isolates were 32 (8–128) µg/mL (Table 3). One isolate (*E. coli*) with co-carriage of *mcr-1* and *mcr-2* exhibited MIC values of 128 µg/mL. The other co-carrying bacterium (*Escherichia coli*) with *mcr-2* and *mcr-3* showed an MIC value of 8 µg/mL. The three *mcr*-positive bacteria that showed phenotypic susceptibilities to colistin sulfate were *Citrobacter portucalensis*, *Citrobacter freundii*, and *Escherichia coli* (Table 3). The median and IQR MIC for *mcr*-negative isolates was 1 (0.5–2) µg/mL, while 20% of the *mcr*-negative isolates (38/190) exhibited resistance to colistin sulfate in agar dilution, with the MIC range from 4 µg/mL to >128 µg/mL (Figure 4).

MIC analyses at each specific value between *mcr*-positive and *mcr*-negative isolates showed that most *mcr*-carrying isolates were identified with higher MIC levels of colistin sulfate, ranging from >8 µg/mL to >128 µg/mL (Figure 4). At the same time, the vast majority of the *mcr*-negative isolates exhibited lower MIC levels, ranging from ≤0.5 µg/mL to 2 µg/mL. A fraction of *mcr*-negative isolates demonstrated MIC values from 32 µg/mL to >128 µg/mL (Figure 4). The overall colistin resistance rate was 31.6% (71/225), with MIC50 and MIC90 at 1 µg/mL and 128 µg/mL, respectively. Conversely, MIC50/MIC90 for the *mcr*-positive and *mcr*-negative subpopulations were 32 µg/mL/128 µg/mL and 1.0 µg/mL/64 µg/mL, respectively.

### 2.5. Multidrug Resistance and mcr-Carriage

All *mcr*-positive isolates (carrying at least one *mcr* gene) were examined for the presence of MDR. The disk diffusion test was conducted to identify whether the 35 *mcr*-positive isolates exhibited susceptibility to the other 17 different antibiotics from 8 different groups or not. Remarkably, 100% of *mcr*-positive organisms exhibited MDR outcomes (Table 4), and *mcr*-negative isolates revealed a comparatively lower frequency of MDR outcomes.

### 2.6. Demographic Factors Associated with mcr-Carriage

More than one type of bacterial isolate was isolated and analyzed from each stool sample. Consequently, 130 bacteria were evaluated from 95 male stool samples and the other 95 from 73 female stool samples. Bacteria isolated from female stool samples carried more proportionate *mcr* genes (16.8%, 16/95) in comparison to male-origin isolates (14.6%, 19/130). In the five different age groups of the study population, almost uniform gene distributions were reported. Overall, the results showed that the sex and age groups were not significantly associated with the presence of *mcr-1*, *mcr-2*, and *mcr-3* (*p* = 0.445 to 0.781) (Table 5).

## 3. Discussion

We believe this is one of the first studies in Bangladesh to investigate colistin-resistant genes in human diarrheal pathogens among infants and children, who comprised an appreciable percentage of participants. Our data clearly showed the association between phenotypic colistin resistance and the *mcr* genes (*mcr-1* to *mcr-5*), similar to other published studies on colistin-resistant bacteria isolated from diarrhea [66,74,75]. This builds on earlier studies in Bangladesh, including among children and adults [60,76].

Isolates harboring *mcr* genes have been detected with high MIC values, showcasing disparities between the agar dilution test and the disk diffusion method [77]. This variation can be attributed to the slow diffusion of colistin disks on agar medium for the complex and large molecular structure of colistin sulfate. In parallel, some *mcr*-negative isolates exhibited resistance to colistin. Several potential reasons may account for this phenomenon. Firstly, mutations in the *mgrB* gene [78], responsible for binding polymyxin antibiotics in the Gram-negative cell wall, are particularly prevalent in *Klebsiella pneumoniae* [79,80]. Secondly, the absence of testing for other variants of *mcr* genes, including *mcr-6*, *mcr-7*, *mcr-8*, *mcr-9*, and *mcr-10*, which could also attribute phenotypic colistin resistance [81,82]. Thirdly, some resistant bacteria may develop capsules, polysaccharide coatings on the outer surface of the cell wall [83,84]. Fourthly, overexpression of efflux pump systems could contribute to resistance development [85,86]. Fifthly, modulation in the bacterial cell surface, including alterations in the structure of lipopolysaccharides (LPSs) of the cell membrane, might affect the binding of polymyxin antibiotics [87]. Additional investigations will be necessary to uncover potential molecular explanations for the observed differences between phenotypic and genotypic colistin resistance, and we will be looking at this more closely in the future.

On the other hand, three isolates appeared phenotypically susceptible to colistin (MIC values 0.5–2.0 µg/mL) in the presence of *mcr-2*/*mcr-1* genes. The observation states that the carriage *mcr* gene variants are not sufficient to confer phenotypic colistin resistance. These findings contradict many earlier reports stating that *mcr* genes provide colistin resistance [34,38,41]. One research identified some genetic substitution mutations, and their reversions are associated with the acquisition and loss of colistin resistance, respectively [88]. The different susceptibilities exhibited could be explained by unstable mobile genetic elements, rare mutations, environmental modulations, and loss of cell membrane LPSs [89,90,91,92]. Comprehensive whole-genome sequencing (WGS) analysis of the bacterial population may decipher the factors that facilitate the evolution of colistin resistance.

In this study, male children had a higher incidence rate of diarrhea than female children, similar to previous studies [93,94]. However, the reason for this difference is unclear. We also found that 57.14% of *mcr*-positive isolates were resistant to amoxicillin/clavulanic acid, and 57 to 71% of *mcr*-carrying isolates were resistant to all generations of cephalosporins, although higher-generation cephalosporins were more susceptible. This is a concern, with clinicians now prescribing more carbapenems due to the decreased potency of cephalosporins, with meropenem showing more susceptibility among the 17 different antibiotics in the eight groups studied. However, recent studies showed carbapenem-resistant *Enterobacteriaceae* are now a global threat [95,96,97]. The MDR status of all *mcr*-positive isolates is positive, which is a threat to public health in Bangladesh and beyond, given the ensuing rise in untreatable infectious diseases [98,99,100]. High MDR associated with *mcr* gene acquisition may influence the pleiotropic resistance of the isolates against a broad group of antimicrobials [101].

Consequently, this study’s findings underscore the significance of establishing comprehensive surveillance systems for priority antibiotics, such as colistin, to help reduce rising AMR rates. Alongside this, we urge the government of Bangladesh to ban the use of colistin as a growth promoter in animal feeds and prophylactically to prevent bacterial infections, similar to other countries [35,36,37]. This is because such measures have been shown to be effective in reducing resistant strains [36,37,53]. Alongside this, instigate antimicrobial stewardship programs (ASPs) across care settings for colistin to other important antibiotics in Bangladesh to reduce resistant strains [102,103,104,105]. This includes ASPs among community pharmacists and drug sellers, building on ASPs in other sectors in Bangladesh [47,106].

We are aware that there are several limitations to this study. Firstly, it was challenging to accurately estimate the real-world scenario since adults experiencing diarrheal problems could readily seek treatment at a healthcare center, whereas children relied on their parents’ decisions and assistance to access medical care. Secondly, the small sample size posed significant obstacles to conducting fully powered statistical analyses, alongside the fact that we undertook convenient sampling. However, our research spanned 15 distinct districts across Bangladesh, and the outcomes are anticipated to be applicable if similar studies are conducted in other districts in the country. Thirdly, while this study examined *mcr* gene variants up to *mcr-5*, newer variants, such as *mcr-6*, *mcr-7*, *mcr-8*, and *mcr-9*, were not investigated. Fourthly, we are aware that a cross-sectional study could not ascertain patients’ health outcomes or treatment outcomes in linkage to *mcr* gene-associated colistin resistance. Having said this, efforts were made to maintain internal validity by conducting independent trials when necessary. Despite these limitations, we believe our findings are robust, providing guidance to all key stakeholders in Bangladesh to enhance future sensitivity to colistin.

## 4. Materials and Methods

### 4.1. Study Design and Sampling

A prospective cross-sectional study was conducted between January 2020 and December 2020 among children and young adults visiting the outpatient department of Uttara Adhunik Medical College Hospital, Dhaka, Bangladesh. A total of 179 children and adults with acute diarrhea participated in this study prior to treatment with any prescribed antibiotics.

Acute diarrhea was defined as three or more liquid, loose, mucous, or bloody stools within 24 h, lasting no longer than 14 days. Fever was defined as a temperature of ≥37.5 °C. Demographic data were obtained from each participant, and informed consent was obtained from the parents or guardians before sample collection. All relevant demographic, clinical, and laboratory data were recorded and transferred to the questionnaire prepared for this study.

The ages of the children and patients were categorized into five groups, namely <1 year, 1–5 years, 6–10 years, 11–15 years, and >15 years, based on previous studies [107]. The income of parents was classified as rich, middle-class, or poor; as we are aware, this can make a difference [108].

A sterilized cotton swab was dipped in the mucous, purulent, or bloody part of the freshly passed stool sample, placed immediately in CaryBlair Medium (Oxoid, Hampshire, UK), and transported to the laboratory for further analysis within six hours of collection.

### 4.2. Isolation and Identification of Bacteria

Collected samples were pre-enriched in buffered peptone water (Oxoid^®^, Hampshire, UK) at a dilution ratio of 1:10 and were incubated overnight at 37 °C. A loopful of each culture was streaked on MacConkey agar (Liofilchem Inc., Roseto degli Abruzzi TE, Italy) and cysteine-, lactose-, and electrolyte-deficient (CLED) agar (Liofilchem Inc., Roseto degli Abruzzi TE, Italy), and subsequently incubated simultaneously at 37 °C for 24 h in aerobic conditions. MacConkey agar supports Gram-negative diarrheal pathogens (Supplementary Figure S1A), while CLED agar aids in the growth of Gram-negative bacteria and Gram-positive cocci, if present in diarrheal samples. Colony counts of 10^3^ or 10^5^ CFU/mL were considered as a cutoff value for a probable diarrheal sample [109].

Gram’s staining and biochemical tests were initially performed to identify growth-positive bacteria. A rapid biochemical test kit API 20E (BioMe’rieux, Durham, NC, USA), consisting of carbohydrate batteries and enzymatic substrates in a set of chromogenic panels, was used to verify the isolated identity (Supplementary Figure S1B) [110]. Part of the bacterial identity was confirmed by the polymerase chain reaction (PCR) amplification and sequencing of the 16S rDNA gene [111]. In total, 228 different isolates were generated from all the diarrheal samples. Three bacteria (one *Proteus* and two staphylococci) were excluded from the study for the next level analyses since colistin resistance is a natural phenomenon of the excluded isolates. The remaining 225 isolates were subjected to an assessment of colistin susceptibility and *mcr-1* to *mcr-5* carriage. The isolates were preserved in 30% glycerol in Trypticase Soy Broth (TSB) at −80 °C until further use.

### 4.3. Phenotypic Colistin Susceptibility Testing

The phenotypic antibiogram profiles of diarrheal isolates against colistin were determined primarily using the Kirby–Bauer disk diffusion method, according to the European Committee on Antimicrobial Susceptibility Testing (EUCAST) and Clinical and Laboratory Standards Institute (CLSI) guidelines (Supplementary Figure S1C) [112,113]. A 3-h bacterial suspension in Mueller–Hinton broth was prepared to a concentration of McFarland 0.5 equivalent and then streaked on Mueller–Hinton agar (MHA, Oxoid, Basingstoke, UK) plates using a cotton swab to ensure consistent growth. The susceptibility of the isolate to the following disks of antibiotics (Oxoid, Basingstoke, UK) was evaluated: colistin (25 µg), amoxycillin + clavulanic acid (30 µg), cefuroxime sodium (30 µg), cefixime (30 µg), cefepime (30 µg), imipenem (10 µg), meropenem (10 µg), nalidixic acid (10 µg), ciprofloxacin (5 µg), lomefloxacin (10 µg), levofloxacin (5 µg), gentamicin (30 µg), amikacin (30 µg), netilmicin (30 µg), tobramycin (10 µg), nitrofurantoin(300 µg), and trimethoprim-sulfamethoxazole (25 µg) by placing them on the bacterial lawns and incubating at 37 °C overnight. A clear zone was developed around the disk for sensitive bacteria, and the zone diameter was measured and evaluated to categorize bacteria as susceptible (S), intermediate (I), and resistant (R) from the CLSI guideline charts for the appropriate antibiotics tested [113].

The isolates’ phenotypic antimicrobial susceptibilities were further tested using the agar dilution method [114]. The lowest concentration of colistin adequate to inhibit the visible growth of bacterial test isolates was determined by minimal inhibitory concentration (MIC) measurement via the agar dilution method [114]. In order to determine the agar dilution MIC, different concentrations of colistin sulfate powder (Santa Cruz Biotechnology Inc., Dallas, TX, USA) from 0.50 µg/mL to 256 µg/mL in a two-fold dilution order were incorporated into the MHA medium accordingly. One pure culture colony was inoculated into Mueller–Hinton broth to prepare each test inoculum. Subsequently, it was incubated for three hours at 37 °C to develop a density of inoculum equivalent to 10^4^ colony-forming units (CFU) per spot to drop on the MHA. The inoculum density was periodically compared to a 0.5 McFarland standard, equivalent to approximately 10^8^ CFU/mL. The plates were incubated at 37 °C in air for 18–20 h. Agar dilution MICs were performed in duplicate. The experiments were repeated when some single colonies were seen or a thin haze growth was observed within the inoculated spot.

The epsilometer test (*E*-test) was performed partly—using a commercial strip containing a predefined gradient of colistin concentrations (Liofilchem Inc., Roseto degli Abruzzi TE, Italy) to validate colistin MIC determination using the agar dilution method [77,115]. Concordant results were found in independent MIC assessments and E-tests (Supplementary Figure S1D,E), as stated earlier [72]. The *E. coli* ATCC 25922 strain was used as the quality control strain for disk diffusion and MIC testing. In addition, a control plate without colistin sulfate was examined for the growth of both test and control isolates. Following EUCAST and CLSI guidelines, isolates were considered susceptible (S) when the MIC values exhibited ≤2 μg/mL and resistant (R) breakpoints when MICs appeared >2 μg/mL [112]. MIC50 and MIC90 values were calculated to report the concentration of colistin that can inhibit the growth of test pathogens by 50%/90%. Multidrug-resistant (MDR) isolates were described as those isolates that were found to be resistant to at least three different classes of antibiotics [116].

### 4.4. Detection of Colistin Resistance mcr Genes

All 225 isolates were subjected to a singleplex polymerase chain reaction (PCR) to detect the *mcr-1* gene, yielding a 309 bp DNA band, using primers described elsewhere [55], and confirmed by sequencing. Amplicons were visualized under UV light after 1.2% agarose gel electrophoresis. The other four primer pairs to detect *mcr-2*, *mcr-3*, *mcr-4*, and *mcr-5* gene amplicons were obtained from a recently published original study [117]. A multiplex polymerase chain reaction (PCR) was conducted to detect the *mcr-1* to *mcr-5* genes in the isolates. In brief, the modified protocol was as follows: prepared bacterial DNA (2 μL) was added to a 2× PCR premixture (15 μL, GeneON, Rhein, Germany), and five pmol of each primer (1 μL), and deionized water was added to obtain a final volume of 30 μL. Reactions went through an initial denaturation at 94 °C for 15 min followed by 25 cycles of amplification (Applied Biosystems 2720 Thermal Cycler, Singapore), consisting of denaturation for 30 s at 94 °C, annealing for 90 s at 55 °C, and extension for 1 min at 72 °C, and a final 10 min elongation at 72 °C. Expected amplicons for *mcr-1* (309 bp), *mcr-2* (715 bp), *mcr-3* (929 bp), *mcr-4* (1116 bp), and *mcr-5* (1644 bp) underwent electrophoresis through 1.2% agarose gel, followed by staining with ethidium bromide, and were visualized under UV light (Supplementary Figure S1F). Lastly, the obtained results were validated by separate singleplex PCR analyses of the *mcr* genes.

### 4.5. Statistical Analysis

Using the IBM SPSS statistics data editor (version 21) and GraphPad prism software (version 9.5), verified data were entered and then examined. The bivariate analysis did not include missing data. The *mcr* gene variations carried by diarrheal pathogens and their phenotypic traits were described using both descriptive and inferential statistical methods. Any associations between categorical data were examined using Pearson’s chi-square test with the appropriate use of Yate’s continuity correction. Fisher’s exact test results of a 2 × 2 contingency table were presented in place of the chi-square test results if the predicted frequency of the test could not be assumed. Two-tailed *p*-values were computed, with a significance level of 0.05.

### 4.6. Ethics Statements

This study was authorized [No. UAMC/ERC/Recommend-62/2018, dated 9 July 2018] by the Ethics Review Committee of Uttara Adhunik Medical College. All research protocols complied with the Declaration of Helsinki regarding the use of human beings in research.

Each adult study participant provided written informed consent before the collection of their urine samples. For patients under the age of 18, parents or legal guardians were asked separately for written informed permission. The patients’ identities were anonymized.

## 5. Conclusions

Our findings indicate that multidrug-resistant pathogenic bacteria containing *mcr* genes are a major reservoir in the guts of young Bangladeshi children and adults. Overall, the *mcr-1*, *mcr-2*, and *mcr-3* variants appear to predominate over other variants, such as *mcr-4* and *mcr-5*, in the diarrheal bacteria of children and adults in Bangladesh. However, newer variants, such as *mcr-6*, *mcr-7*, *mcr-8*, and *mcr-9*, were not investigated in this study. Overall, we did not find any association between phenotypic colistin resistance and age or sex.

The advent of clinical MDR pathogens resistant to colistin, which is designated as an antibiotic of last resort, is a growing concern in Bangladesh and other countries, as it leads to infectious diseases that are subsequently difficult to treat. These findings require immediate monitoring and action from the government and other key stakeholders in Bangladesh in line with the goals of the NAP. Proposed activities include additional research activities to uncover potential molecular explanations for the observed differences between phenotypic and genotypic colistin resistance, as well as limiting the use of colistin as a growth promoter agent in animal husbandry in Bangladesh, similar to other countries. Concerted effort is especially important, as AMR is increasingly being transferred from animals to humans as a result of improper antibiotic use in animal feeding. Alongside this, there is a need to instigate the active monitoring of colistin resistance and the resistance patterns of other important antibiotics, which is also in line with the goals and activities of the Bangladesh NAP. There also needs to be increased patient education to address concerns with hygiene levels in the country, as well as instigating measures to improve the availability of safe drinking water. Other important activities in Bangladesh include instigating pertinent ASPs in ambulatory care to help physicians, pharmacists, and drug sellers reduce inappropriate prescribing and dispensing of colistin. This builds on successful ASPs in ambulatory care in other LMICs. We will continue to monitor the situation.

## Figures and Tables

**Table 1:** Identified diarrheal pathogens carrying *mcr* gene variants ^a^.

Bacteria Type ^b^	Number of Isolates Carrying *mcr* Genes		Percentage of *mcr*-Positive Isolates
	Positive	Negative	
*Escherichia* spp. ^c^	20	120	14.3
*Shigella flexneri*	5	8	38.5
*Citrobacter* spp. ^d^	5	15	25
*Klebsiella pneumoniae*	2	16	11.1
*Enterobacter hormaechei*	2	8	20
*Pseudomonas parafulva*	1	2	33.3
*Aeromonas caviae*	0	3	-
*Acinetobacter* spp.	0	2	-
*Bacillus cereus*	0	1	-
*Bacterium endosymbiont*	0	1	-
*Morganella morganii*	0	3	-
*Serratia marcescens*	0	1	-
*Stenotrophomonas maltoph*	0	7	-
*Vibrio neocaledonicus*	0	1	-
*Cronobacter sakazakii*	0	1	-
*Enterococcus faecium*	0	1	-
Total	35	190	

Note: ^a^ 12 *mcr-1* and two *mcr-2* genes were identified by polymerase chain reaction (PCR). ^b^ Bacteria were primarily identified based on their growth on selective culture media, followed by biochemical tests. Further, identifications were confirmed by API 20E and 16s rDNA sequencing. ^c^ *Escherichia* spp. includes three *Escherichia fergusonii* and 17 *Escherichia coli*. ^d^ *Citrobacter* spp. includes one *Citrobacter europaeus*, two *Citrobacter portucalensis*, and two *Citrobacter portucalensis*.

**Table 2:** Phenotypic genotypic association of *mcr* genes and colistin resistance.

Presence of *mcr* Gene Varients ^a^		Phenotypic Susceptibility ^b^		*p* Value
		Sensitive	Resistance	
*mcr-1*	Positive (7)	1	6	0.001
	Negative (218)	155	63	
*mcr-2*	Positive (17)	3	14	0.000
	Negative (208)	153	55	
*mcr-3*	Positive (13)	0	13	0.000
	Negative (212)	156	56	
combined	Positive (35)	3	32	0.000
	Negative (190)	152	38	

Note: ^a^ Two bacterial isolates were co-carrying two different *mcr* genes. ^b^ Phenotypic colistin resistance was measured using the minimum inhibitory concentration (MIC) breakpoint measurement by the agar dilution method.

**Table 3:** Diarrheal isolates with identified *mcr*-gene variants and associated minimum inhibitory concentration of colistin.

*mcr*-Possitive Isolate ID	Identified Bacteria ^a^	Identified *mcr* Gene Varient	Phenotypic Colistin Susceptibility by MIC (µg/mL) ^b^
PBD009	*Shigella flexneri*	*mcr-3*	256
PBD014	*Klebsiella pneumoniae*	*mcr-2*	128
PBD018	*Escherichia coli*	*mcr-3*	8
PBD021	*Escherichia coli*	*mcr-3*	128
PBD022	*Escherichia coli*	*mcr-3*	32
PBD027	*Citrobacter portucalensis*	*mcr-2*	0.5
PBD028	*Escherichia coli*	*mcr-3*	256
PBD033C2	*Pseudomonas parafulva*	*mcr-1*	128
PBD35	*Citrobacter portucalensis*	*mcr-2*	8
PBD35C1	*Citrobacter freundii*	*mcr-2*	128
PBD35C2	*Citrobacter freundii*	*mcr-2*	1
PBD039	*Escherichia fergusonii*	*mcr-2*	256
PBD040	*Citrobacter europaeus*	*mcr-2*	64
PBD043	*Klebsiella pneumoniae*	*mcr-2*	16
PBD058	*Escherichia coli*	*mcr-2*, *mcr-3*	8
PBD062	*Escherichia fergusonii*	*mcr-2*	32
PBD072	*Escheril chia fergusonii*	*mcr-2*	64
PBD077	*Escherichia coli*	*mcr-1*	8
PBD077C1	*Shigella flexneri*	*mcr-1*	32
PBD077C2	*Escherichia coli*	*mcr-1*, *mcr-2*	128
PBD080C2	*Escherichia coli*	*mcr-1*	2
PBD081C3	*Escherichia coli*	*mcr-2*	32
PBD081C4	*Enterobacter hormaechei*	*mcr-3*	128
PBD081C1	*Escherichia coli*	*mcr-2*	32
PBD082	*Shigella flexneri*	*mcr-1*	128
PBD083C1	*Shigella flexneri*	*mcr-3*	32
PBD083C2	*Escherichia coli*	*mcr-2*	64
PBD084C1	*Enterobacter hormaechei*	*mcr-2*	128
PBD84C2	*Escherichia coli*	*mcr-2*	4
PBD090	*Escherichia coli*	*mcr-3*	64
PBD096	*Escherichia coli*	*mcr-3*	8
PBD107	*Escherichia coli*	*mcr-1*	8
PBD114	*Escherichia coli*	*mcr-3*	8
PBD116	*Escherichia coli*	*mcr-3*	64
PBD117	*Shigella flexneri*	*mcr-3*	64

Note: ^a^ Bacteria were identified using the rapid biochemical test kit API 20E system (BioMe’rieux, Durham, NC, USA) followed by16s rDNA sequencing. ^b^ The minimum inhibitory concentration (MIC) measurement was conducted by the agar dilution method following the EUCAST guidelines.

**Table 4:** Multidrug resistance (MDR) phenomena of *mcr*-positive isolates.

List of Antibiotics Tested (*n* = 17, from Eight Drug-Classes)		Phenotypic Susceptibilities of *mcr*-Positive Diarrheal Isolates																	
Drug Class	Antibiotic Name	PBD009	PBD014	PBD018	PBD021	PBD022	PBD027	PBD028	PBD033C2 ^b^	PBD035	PBD035C1	PBD035C2	PBD039	PBD040	PBD043	PBD058	PBD062	PBD072	PBD077
β-lactam with β-lactamase inhibitor	Amoxi-clav ^a^	R	R	R	R	R	R	I	R	R	R	S	R	I	R	I	S	S	S
Cephalosporins	Cefuroxime-G2 ^c^	R	R	R	R	I	I	I	R	R	R	R	R	R	R	S	R	R	S
	Cefixime-G3	R	R	R	R	I	R	R	R	R	R	R	R	R	R	S	R	R	S
	Cefepime-G4	R	R	R	R	R	I	R	R	I	I	I	R	I	R	I	R	R	S
Carbapenems	Imipenem	R	R	I	R	R	I	I	R	I	R	S	I	I	R	S	I	R	S
	Meropenem	R	R	S	R	R	S	R	S	S	S	S	I	S	S	S	S	R	S
Quinolone and fluoroquinolones	Nalidixic acid	I	S	R	I	I	I	I	R	I	R	R	R	I	R	R	R	R	R
	Ciprofloxacin	R	S	R	S	S	S	R	R	I	R	R	R	R	S	I	R	I	R
	Levofloxacin	R	S	R	S	S	S	I	R	S	I	S	R	S	R	S	R	S	R
	Lomefloxacin	S	S	R	S	I	S	R	R	I	R	R	R	S	I	R	R	R	R
Aminoglycosides	Gentamicin	S	S	I	S	R	S	I	S	S	S	S	S	S	R	S	R	R	R
	Amikacin	R	S	I	S	S	S	R	S	S	I	I	R	I	R	S	I	I	I
	Netilmicin	S	S	I	I	S	S	R	S	S	S	I	I	R	S	R	S	R	I
	Tobramycin	S	S	S	S	R	S	R	I	S	S	S	R	S	R	S	R	R	S
Polymyxins	Colistin	R	S	R	S	R	R	R	R	S	R	R	R	R	R	S	S	R	R
Nitrofuran	Nitrofurantoin	I	R	I	R	R	I	R	R	S	I	S	S	I	R	I	R	I	I
Trimethoprim	Trimethoprim-sulfamethoxazole	S	S	R	S	R	I	S	R	S	R	R	S	S	S	R	R	R	R
MDR status ^d^		+	+	+	+	+	+	+		+	+	+	+	+	+	+	+	+	+

Note: R, Resistant; S, Sensitive; I, Intermediate; MDR, multidrug-resistant. ^a^ Amoxicillin-clavulanic acid. ^b^ PBD033C2 is *Pseudomonas* spp., which are naturally resistant to amoxicillin-clavulanic acid; therefore, AST results have been excluded from the MDR calculation. PBD081C4 and PBD084C1 are *Enterobacter* spp., which are naturally resistant to amoxicillin-clavulanic acid; therefore, AST results have been excluded from the MDR calculation. ^c^ Different generations of cephalosporin. ^d^ Multidrug-resistant isolate is defined when an isolate becomes resistant to at least one antibiotic in three or more drug classes [73]. ‘+’ indicates MDR-positive.

**Table 5:** Demographic factors associated with *mcr* genes (*n* = 225).

Demography		Number (%) of Different *mcr*-Gene Variants		*p*-Value
		***mcr-1* Positive** **(*n* = 7)**	***mcr-1* Negative** **(*n* = 218)**	
Gender	Male	5 (3.8)	125 (96.2)	0.702 *
	Female	2 (2.1)	94 (97.9)	
Age group (years)	<1	4 (4.6)	83 (95.4)	0.707 *
	1–5	3 (3.3)	88 (96.7)	
	6–10	0	12 (100)	
	11–15	0	5 (100)	
	>15	0	30 (100)	
		***mcr-2* positive** **(*n* = 17)**	***mcr-2* negative** **(*n* = 208)**	
Gender	Male	8 (6.2)	122 (93.8)	0.445 *
	Female	9 (9.5)	87 (90.5)	
Age group (years)	<1	8 (9.2)	79 (90.8)	0.480 *
	1–5	4 (4.4)	87 (95.6)	
	6–10	1 (8.3)	11 (91.7)	
	11–15	0	5 (100)	
	>15	4 (13.3)	26 (86.7)	
		***mcr-3* positive** **(*n* = 13)**	***mcr-3* negative** **(*n* = 212)**	
Gender	Male	7 (5.4)	123 (94.6)	0.779 *
	Female	6 (6.3)	89 (93.7)	
Age group (years)	<1	5 (5.7)	82 (94.3)	0.781 *
	1–5	4 (4.4)	87 (87)	
	6–10	1 (8.3)	11 (91.7)	
	11–15	0	5 (100)	
	>15	3 (10)	27 (90)	
		***mcr-1* to *mcr-3* positive** **(*n* = 35)**	***mcr-1* to *mcr-3* negative** **(*n* = 190)**	
Gender	Male	19 (14.6)	111 (85.4)	0.711 *
	Female	16 (16.8)	79 (83.2)	
Age group (years)	<1	15 (17.2)	72 (82.8)	0.503 *
	1–5	11 (12.1)	80 (87.9)	
	6–10	2 (16.7)	10 (83.3)	
	11–15	0	5 (100)	
	>15	7 (23.3)	23 (76.7)	

Note: %, row percentage; * Fisher’s exact test.

## Data Availability

Data are contained within the article and available upon request.

## Supplementary Materials

The following supporting information can be downloaded at: https://www.mdpi.com/article/10.3390/antibiotics13060534/s1. The supporting information on detailed procedures for bacterial isolation, antibiotic susceptibility testing, and *mcr* detection via Polymerase Chain Reaction (PCR), as outlined in Supplementary Figure S1.
