# Supplementary material for: Mobile Colistin-Resistant Genes mcr-1, mcr-2, and mcr-3 Identified in Diarrheal Pathogens among Infants, Children, and Adults in Bangladesh: Implications for the Future
Source: Antibiotics (Basel). 2024 Jun 7;13(6):534. doi: 10.3390/antibiotics13060534 (PMC11200974; doi:10.3390/antibiotics13060534)
Supplement: Supplementary file 1 [file antibiotics-13-00534-s001.zip › antibiotics-2959398-supplementary.pdf]

The Supplementary Data for-

Mobile Colistin-Resistant Genes mcr-1, mcr-2, and mcr-3 Identified in Diarrheal Pathogens among Infants, Children, and Adults in Bangladesh: Implications for the Future

Shafiuzzaman Sarker 1, Reeashat Muhit Neeloy 1, Marnusa Binte Habib 1, Umme Laila Urmi 1,2, Mamun Al Asad 1, Abu Syed Md. Mosaddek 3, Mohammad Rabiul Karim Khan 4, Shamsun Nahar 1, Brian Godman 5,6 and Salequl Islam 1,2,*

1 Department of Microbiology, Jahangirnagar University, Savar, Dhaka 1342, Bangladesh
2 School of Optometry and Vision Science, UNSW Sydney, Sydney, NSW 2052, Australia
3 Department of Pharmacology, Uttara Adhunik Medical College, Dhaka 1230, Bangladesh
4 Sheikh Hasina National Institute of Burn and Plastic Surgery (SHNIBP), Dhaka 1000, Bangladesh
5 Strathclyde Institute of Pharmacy and Biomedical Sciences, University of Strathclyde, Glasgow G4 0RE, UK
6 Division of Public Health Pharmacy and Management, School of Pharmacy, Sefako Makgatho Health Sciences University, Pretoria 0204, South Africa
* Correspondence: salequl@juniv.edu; Tel.: +880-1715029136; Fax: +880-2-77910

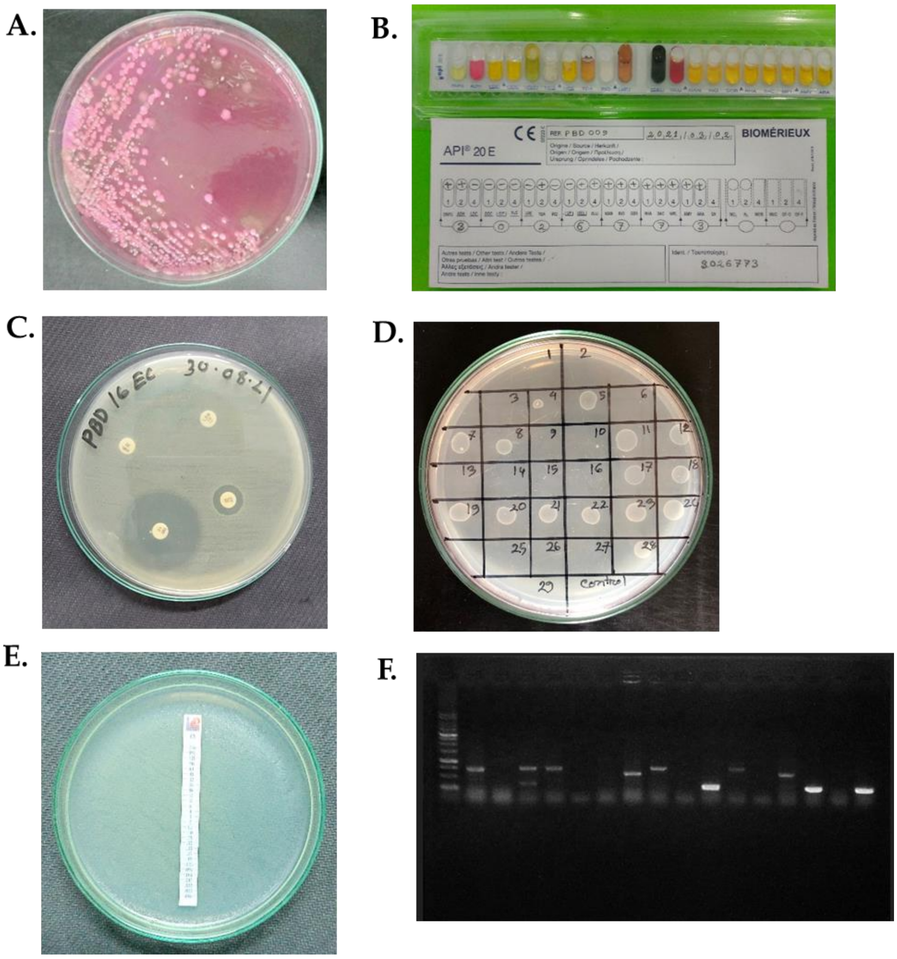

Figure S1: Bacterial Isolation, Antibiotic Susceptibility Testing, and mcr Detection by Polymerase Chain Reaction (PCR).

A) Pre-enriched diarrheal samples were plated onto MacConkey agar medium, and visible bacterial growth was observed following overnight incubation at 37°C.

B) Further confirmation of bacterial identification was conducted using API E20 strips.

C) Antibiotic susceptibility testing via disk diffusion involved placing a colistin sulfate disk on the bacterial lawn, followed by measurement of zone diameter after 18-24 hours of incubation at 37°C.

D) The agar dilution method entailed dissolving colistin sulfate powder in sterile distilled water and adding it to molten MH agar to create 2-fold dilutions, resulting in final concentrations ranging from 0.5 µg/mL to 256 µg/mL. Multiple spots were manually divided on one petri dish MHA plate, with each spot receiving 1 µL of a bacterial inoculum containing approximately 10^4 CFU of bacteria. Plates were then incubated for 18-20 hours at 37°C. Phenotypic resistance to colistin sulfate, according to European Committee on Antimicrobial Susceptibility Testing (EUCAST) guidelines, was determined by visible bacterial growth on plates containing >2 µg/mL colistin sulfate. Additionally, this method determined the minimal inhibitory concentration (MIC) as the lowest concentration of colistin capable of inhibiting visible bacterial growth.

E) MIC measurement was performed using the commercial Etest (Liofilchem Inc, Italy).

F) Amplified PCR products of *mcr-1* to *mcr-5* genes were subjected to electrophoresis on a 1.2% agarose gel and visualized under UV light. Lane 1 displays a 1000 base pair DNA marker (Gene ruler, Thermo Fisher Scientific, MA).
